# Editorial: Anti-Inflammatory Agents in the Context of Age-Related Cardiometabolic Disease: Ethnopharmacological Perspectives

**DOI:** 10.3389/fphar.2022.923287

**Published:** 2022-06-24

**Authors:** Somasundaram Arumugam, Wawaimuli Arozal, Koji Ikeda

**Affiliations:** ^1^ Department of Pharmacology and Toxicology, National Institute of Pharmaceutical Education and Research (NIPER)-Kolkata, Kolkata, India; ^2^ Department of Pharmacology and Therapeutics, Faculty of Medicine, Universitas Indonesia, Jakarta, Indonesia; ^3^ Department of Epidemiology for Longevity and Regional Health, Kyoto Prefectural University of Medicine, Kyoto, Japan

**Keywords:** cardiometabolic disease, aging, senescence, ethnopharmacology, inflammation

Aging is a normal physiological process altering cell function largely through cellular senescence, but sometimes accelerated aging and/or excess accumulation of senescent cells in individuals may lead to several complications affecting the quality of life. One such complication is cardiometabolic disease, which is commonly seen in the elderly and is a global burden, causing health and economic problems. Though various treatment strategies are made available to manage the cardiometabolic diseases such as atherosclerosis, angina pectoris, and myocardial infarction (MI), etc., traditional systems of medicine play a major role in their treatment in various countries, such as Ayurveda, traditional Chinese medicine (TCM), Japanese Kampo medicine, Homoeopathy, Unani, and Siddha, etc. The lack of evidence for their beneficial effects as well as their molecular effects urged us to choose this topic. Louisa et al. from the University of Indonesia have performed a systematic review on the role *of Moringa oleifera* Lam [Moringaceae] in cardiovascular or metabolic disorders, in addition to its high nutritional value. *Moringa oleifera* Lam. is a native plant of several Asian countries and is reported to provide potential benefits against oxidative stress and inflammation, modulating glucose and lipid metabolism and preventing end-organ damage. They have also covered the importance of altering the gut microbiota in metabolic syndrome in addition to the supporting data for the role of *Moringa oleifera* Lam. on various signaling pathways activated during inflammation and oxidative stress. Liu et al. have studied the molecular basis of the effect of *ShenLian* extract on atherosclerotic plaques *in vitro* and *in vivo*. By using the co-culture model, macrophage and smooth muscle cell (SMC) interactions were studied and reported the necessary role of transforming growth factor (TGF)-β in the cross-talk between macrophages and SMC in stabilizing the atherosclerotic plaques. They have reported that *ShenLian* extract (an herbal decoction consisting of *Coptis chinensis* Franch. and *Panax ginseng* C.A.Mey.) could stabilize the vulnerable plaques by increasing plaque collagen and functionally reconstruct the extracellular matrix via increasing TGF-β expression and regulating the STAT3/SOCS3 pathway. Liu et al. and her team reported that the monocyte locomotion inhibitory factor (MLIF), a heat-stable pentapeptide from *Entamoeba histolytica*, promoted microglia transition toward the M2 phenotype, which contributes to neuronal survival and tissue repair, *in vivo* and *in vitro* ischemic stroke model via regulation of eEF1A1/NFκB signaling pathway. They have suggested that MLIF might be a useful pharmacological agent for ischemic stroke. Zhang et al. have performed a meta-analysis on the systematic evaluation of TCM in the treatment of ventricular remodeling after acute MI. They have analyzed various parameters of myocardial function, ventricular remodeling, and serum levels of BNP and CRP from forty randomized clinical trials involving 3,659 participants and proved that a combination of TCM or TCM preparations with conventional Western medicine could prevent and reverse ventricular remodeling in post-acute MI (AMI). They have concluded that the combination of TCM and Western medicine can alleviate ventricular remodeling, enhance cardiac function, and reduce the incidence of major adverse cardiac events in AMI patients. Though fewer articles are available on this topic, we hope that this Research Topic provides readers with the importance of ethnopharmacology and traditional medicine system in the treatment of cardiometabolic diseases, initiates new ideas, and enhances future research to advance the field.

